# Turkish version of impact on family scale: a study of reliability and validity

**DOI:** 10.1186/1477-7525-7-4

**Published:** 2009-01-28

**Authors:** Nilgun Bek, I Engin Simsek, Suat Erel, Yavuz Yakut, Fatma Uygur

**Affiliations:** 1 Department of Physical Therapy and Rehabilitation, Faculty of Health Sciences, Hacettepe University, Ankara, Turkey

## Abstract

**Background:**

Although there is a considerably high prevalence of developmental disorders in Turkey there are not many assessment tools related to evaluating the impact of these children on their family. The aim of this study was to determine the validity and reliability of the Turkish version of the Impact on Family Scale (IPFAM), a health related quality of life measurement to be utilized in clinical trials, health care services, research and evaluation.

**Methods:**

Caregivers of 85 children with developmental disabilities answered the questionnaire and 65 of them answered it twice with a one week interval. The reliability of the measurement was assessed by Cronbach's alpha coefficient, and with intraclass correlation coefficient (ICC) for test-retest reliability. Construct validity was assessed by calculating the correlation between total impact score of IPFAM, WeeFIM and the physiotherapists' evaluation via Visual Analogue Scale (VAS) to determine the child's disability.

**Results:**

Test-retest reliability was found to be ICC = 0.953 for total impact, 0.843 for financial support, 0.940 for general impact, 0.871 for disruption of social relations and 0.787 for coping. Internal consistency was tested using Cronbach's alpha and was found to be 0.902 for total impact of IPFAM. For construct validity the correlation between total impact score of IPFAM and WeeFIM was r = -0,532 (p < 0.001) and the correlation between total impact score of IPFAM and the physiotherapist's evaluation was r = 0.519 (p < 0.001).

**Conclusion:**

The Turkish version of IPFAM was found to be a reliable and valid instrument for assessing the impact of developmental disorders of the child on the family.

## Background

Within the cost conscious healthcare system, health-related quality of life measurement has become an important health outcome. This is most obvious in current multi-centered studies [1]. However, while assessing the quality of life amongst the disabled, the burden carried by their caregivers is usually not given adequate consideration. This is particularly true for the families of impaired children. Because the extent to which the family is burdened by the child's illness may also influence child's own quality of life.

Chronic childhood illnesses have considerable consequences for the family system. Thus, the process of family adaptation to the presence of an ill member has been explored in the medical and sociological literature [2,3]. Various studies have been carried out examining family burden associated with psychiatrically disturbed youth [4-6]. Researchers have stated that understanding the experience of family burden in this population is crucial since the emotional, social and financial cost of living with a disturbed youngster may affect the mental health of family members, while playing a role in decisions to seek out and use children's mental health services [4]. Moreover, Riley et al. states that this line of research enables decision makers to compare different treatments not only in terms of costs but also valued outcomes [7].

These statements also hold true for children with developmental disorders such as cerebral palsy, meningomyolecel etc.; because whether the problem is a chronic illness, a psychiatric disorder or a developmental disorder, there will be an impact on the families' daily routines.

Stein and Riessman have developed the Impact on Family Scale which was designed to measure the impact of pediatric chronic health conditions on parents and family. They published their preliminary findings in 1980 [3]. In their study, impact was defined as the effects of a child's illness on the family system. The implicit assumption was that changes occur in the family because of illness, forcing adaptations in the family environment [3]. The Impact on Family Scale is a 27-item inventory that takes approximately 10 minutes to complete and can be used either as a questionnaire, when reading levels are adequate, or an interviewer-administered form [8]. Five dimensions are assessed: 1) economic burden or the extent to which the illness changes the economic status of the family, 2) social impact, or the quality and quantity of interaction with others outside the immediate family, 3) familial impact, the quality of interaction within the family unit, 4) personal strain, subjective burden experienced by the primary caregiver, and 5) Coping, strategies employed by the family to master the stress of an illness or disability of the child. All items have a standard four point scale ranging from strongly agree to strongly disagree.

As in the whole world, there is an increasing interest to assess health related quality of life in Turkey. Although there is considerably high chronic childhood disease prevalence, especially muscle diseases due to marriage among first degree relatives such as cousins, there are not many assessment tools related to children in Turkish. So far, the Turkish versions of the Childhood Health Assessment Questionnaire (CHAQ), the Child Health Questionnaire (CHQ) and Functional Independence Measure of Children (WeeFIM) have been studied for reliability and validity [1,9-11].

Direct translation of questionnaires into other languages does not guarantee maintenance of validity. It is well recognized that if measures are to be used across cultures, the items must not only be well translated linguistically, but also adapted culturally in order to maintain the content validity of the instrument across different cultures [12].

The purpose of this study was to develop the Turkish version of the Family Impact Scale and to examine whether it is a valid and reliable tool for assessing the impact of having a child with chronic disability on family life.

## Methods

### Procedure

This study is divided into two phases: Phase I, the cross-cultural adaptation, which involves the translation procedures and preliminary probe in the target population; and phase II, which involves the reliability and validation study. Phase I: Cross cultural adaptation process followed the guidelines provided by Guillemin et al, Beaton et al. and Ruperto et al. [[1,13], and [14]]. Two forward translations were carried out by independent translators from English to their native language which is Turkish. A meeting was then convened among the two forward translators and four other physiotherapists not involved in the translation procedures but who were experienced in treating children with chronic disabilities. The goal of this meeting was to reach a consensus among the members of the group to obtain the first unified version of the two forward translations. This version was then back translated by two independent translators whose native language was English. They were fluent into the idioms and colloquial forms of the forward language. The two back translators had not seen the original English text of the Family Impact Scale and were unaware of the purpose of the project. The two backward translations were then reviewed by two of the authors of this paper. The aim of this phase was to ascertain that the translation was fully comprehensible and a concordance with the English version was attained. Following this phase a second meeting was held with participation of all the interested professionals. The purpose of this meeting was to reach a final consensus. To ensure that the adapted version still retains its equivalence in an applied situation, the last stage of the adaptation process was to test the pre-final version in a pilot study. A health professional experienced in treating chronic childhood diseases administered the questionnaire to the parents, asking them to consider each question in a critical manner and judged whether the questions were understood. The only problematic item in this stage was "sometimes I fell like we live on a roller coaster..." because roller coaster did not convey an appropriate meaning for the Turkish population. This word was replaced with another descriptor conveying the same meaning. This version was finalized with consensus of a bilingual team experienced in treating children with chronic disabilities as advocated by former researchers [[1,13], and [14]].

The scoring of the IPFAM was done according to the scoring instructions given in the PACTS PAPERS/AECOM [15]. The results obtained were computed under the headings Total Impact, Financial Support, General Impact, Disruption of Social Relations and Coping for statistical analysis. Although IPFAM originally had 27 items, due to precise scoring instructions, we used 24 items and the revised scoring based on PACTS data. We recoded the given items to the opposite; so that low impact had the lower score. However, total impact is not the mere sum of the 24 items. It does not include the items with a positive implication which did not require to be recoded to the opposite direction.

### Participants

Informed consent was obtained from all subjects and ethical approval was obtained from the University's Ethics Committee.

The caregiver parent of 85 children between the ages of one and nine years (mean ± standard deviation = 6.52 ± 3.33 years) participated in the study. Eighty-five children of which 50 (59%) were with a diagnosis of cerebral palsy, 23 (27%) with muscular disease, 6 (7%) with mental motor retardation, 4 (5%) with spina bifida, 2 (2%) with meningitis, were recruited among the patients receiving physiotherapy in a University Hospital who volunteered to take part in the study, so they constituted a sample of convenience. No inclusion criterion was identified except having at least one child with a disability who is one year old or older. The participants were asked to answer the questionnaire for a second time, after an interval of one week. Relevant socio-demographic data were given in Table 1.

**Table 1:** Demographic data's of participants.

	**X ± SD**
**Age (children) (years)**	6.52 ± 3.33
**Education level of parents (years)**	
Mother	7.95 ± 3.77
Father	9.33 ± 3.74
	**n (%)**
**Health insurance **	
With	79 (93)
Without	6 (7)
**Marital status**	
Married	82 (96)
Divorced	3 (4)
**Residence**	
Living with parent	85 (100)

### Data analysis

There were no missing values for the test or retest of the items of IPFAM. However, 20 parents were not able to complete the retest due to unexpected health problems, vacations, and because they lived in other cities and came for treatment on a bimonthly or monthly basis. The completion duration of the test was between 8 to 12 minutes

#### Reliability

Cronbach's alpha was used to assess the internal consistency of the IPFAM. Also, subscales to total and inter-subscale, correlations were used to evaluate internal consistency with Pearson correlation analysis. Test-retest values of subgroups and total scores were compared with the Wilcoxon signed rank test (two-tailed). The test-retest reliability was calculated on the answers of 65 parents who were able to complete the questionnaire twice with an interval of one week by using intra-class correlation coefficient (ICC).

#### Validity

Construct validity was evaluated by hypothesizing how the measure should behave and confirming or disconfirming this hypothesis. Thus, construct validity was investigated through an analysis of the intercorrelations among the items with the benchmark criterion. One of the benchmarks was the physiotherapist's evaluation of disability intensity on a 10 cm visual analogue scale (VAS) anchored with negligible disability to total disability. The physiotherapist who carried out the evaluation of disability had specialized in pediatric rehabilitation and had been treating the child for at least three months. Construct validity was also measured by comparing the IPFAM responses with the results of The Functional Independence Measure for Children (WeeFIM). WeeFIM is an 18 item, 7 level ordinal scale instrument that measures a child's consistent performance in essential daily functional skills such as self-care, sphincter control, transfers, locomotion, communication and social cognition under three main domains which are self-care, mobility and cognition [16,17]. Our choice of using WeeFIM was based on the age span of our children with developmental disabilities and on the assumption that functional independence of a child is directly proportional to the burden of care affecting the family. We chose WeeFIM also because the reliability and validity of the Turkish version has been demonstrated [11].

All statistical analysis was done with SPSS 10.0 for Windows. A probability value of p < 0.05 was considered to indicate a significant effect.

## Results

The means and standard deviations of the five dimensions of IPFAM, the physiotherapists' assessment and WeeFIM are shown in Table 2. To enable comparisons, we standardized the scores by dividing each subscale score by the number of items used to produce it (Table 3).

**Table 2:** Data related to the IPFAM dimensions, WeeFIM and physiotherapist's assessment.

	**Test** **X ± SD**	**Retest** **X ± SD**
**Total Impact**	52.02 ± 12.09	51.74 ± 11.62
**Financial Support**	8.69 ± 2.41	8.68 ± 2.33
**General Impact**	26.95 ± 6.29	26.70 ± 6.07
**Disruption of Social Relation**	22.15 ± 6.05	22.04 ± 5.74
**Coping**	6.47 ± 2	6.74 ± 2.05
**Physiotherapist's Assessment (VAS mm)**	44.27 ± 28.82	-
**WeeFIM**	72.17 ± 41.56	-

**Table 3:** Data related to the IPFAM dimensions with standardized scores

	**Number of items**	**Test** **X ± SD**	**Retest** **X ± SD**
**Total Impact**	19	2.74 ± 0.64	2.72 ± 0.61
**Financial Support**	3	2.90 ± 0.80	2.89 ± 0.78
**General Impact**	10	2.70 ± 0.63	2.67 ± 0.61
**Disruption of Social Relation**	9	2.46 ± 0.67	2.45 ± 0.64
**Coping**	4	1.62 ± 0.50	1.69 ± 0.51

According to the Wilcoxon signed rank test, there was no difference between test and retest values of the total impact, financial support, general impact, disruption of social relations and coping (p < 0.05).

Inter-subscale correlations were found to be between r = -0,016 and r = 0,851. However coping subscale was not significantly correlated to any other subscales. Thus, not surprisingly when coping was excluded inter-subscale correlations were calculated as between r = 0,637 and r = 0,851 (p < 0.05).

The same was also true for subscale to total correlation analysis. The correlations between subscales and total impact score ranged between r = -0,107 and r = 0,957. There was no correlation between coping and total impact. The average subscale to total impact correlation was 0.875 when coping was excluded (Table 4).

**Table 4:** Inter-subscale and subscale – total correlation matrix for test.

	**Total Impact**	**Financial Support **	**General Impact**	**Disruption of Social Relations**	**Coping**
	**r (p)**	**r (p)**	**r (p)**	**r (p)**	**r (p)**
**Total Impact**	-				
**Financial Support**	0.742 (0.000)*	-			
**General Impact**	0.957 (0.000)*	0.670 (0.000)*	-		
**Disruption of Social Relations**	0.925 (0.000)*	0.637 (0.000)*	0.851 (0.000)*	-	
**Coping**	-0.107 (0.332)	-0.086 (0.435)	-0.036 (0.742)	-0.016 (0.886)	-

*: p < 0.01.

### Reliability

For internal consistency reliability analysis, Cronbach's alpha was calculated to be 0,902 for total impact score (Table 5).

**Table 5:** Reliability Analysis Cronbach's Alpha.

	**Number of items**	**Alpha Coefficient**
**Total Impact**	19	0.902
**Financial Support**	3	0.715
**General Impact**	10	0.796
**Disruption of Social Relation**	9	0.825
**Coping**	4	0.439

Test-retest reliability was found to be ICC = 0.953 for total impact, 0.843 for financial support, 0.940 for general impact, 0.871 for disruption of social relations and 0.787 for coping (Table 6).

**Table 6:** Test – retest reliability.

	**ICC**	**(95% Confidence Interval)**
**Total Impact**	0.953	(0.928–0.969)
**Financial Support**	0.843	(0.767–0.895)
**General Impact**	0.940	(0.909–0.961)
**Disruption of Social Relation**	0.871	(0.807–0.914)
**Coping**	0.787	(0.690–0.856)

### Validity

Correlation between the total impact score of IPFAM and WeeFIM, and between the total impact score of IPFAM and physiotherapists' evaluation via VAS were tested for construct validity. The resulting correlation was r = -0.532 (p < 0.001) for WeeFIM and 0.519 (p < 0.001) for VAS (Table 7). Correlations between IPFAM and WeeFIM at subscale level showed good construct validity except coping subscale (Table 8).

**Table 7:** Construct validity.

	**Total Impact**
	**r (p)**
**Physiotherapist's Assessment (VAS)**	0.519 (0.000)
**WeeFIM**	-0.532 (0.000)

**Table 8:** Correlation between IPFAM subscales and WeeFIM subscales.

	**Total WeeFIM**	**WeeFIM Self-Care**	**WeeFIM Mobility**	**WeeFIM Cognition**
	**r (p)**	**r (p)**	**r (p)**	**R (p)**
**Total Impact**	-0.532 (0.000)*	-0.532 (0.000)*	-0.522 (0.000)*	-0.447 (0.002)*
**Financial Support**	-0.496 (0.000)*	-0.439 (0.002)*	-0.451 (0.002)*	-0.450 (0.002)*
**General Impact**	-0.464 (0.000)*	-0.516 (0.000)*	-0.490 (0.001)*	-0.394 (0.007)*
**Disruption of Social Relations**	-0.472 (0.001)*	-0.446 (0.002)*	-0.487 (0.001)*	-0.409 (0.005)*
**Coping**	-0.058(0.695)	0.017 (0.911)	-0.094 (0.536)	0.036 (0.814)

*: p < 0.05

## Discussion

Despite the significant social and emotional costs of caring for an ill or disabled child, the majority of studies aiming to measure the impact on families are mainly developed in the English language.

A translation on which a consensus was obtained was our first objective. In our study, the Turkish version did not require any changes except for the concept of 'roller coaster' in place of which a phrase was found conveying the same meaning. Consequently, it was concluded that the questionnaire was easily comprehensible to the Turkish population.

The absence of missing values in the test may be due to the fact that the respondents constituted a sample of convenience from the parents whose children were receiving routine physiotherapy. Consequently they may have felt obliged to answer the whole questionnaire. We believe this may be a weakness of the study, since response rates may not hold true for a general population.

When data related to IPFAM dimensions are observed in Tables 2, 3 and 4, the average inter-subscale and subscale to total impact correlations were found to be higher when coping was excluded. These results indicate that although it may give valuable information about a family's ability to master the daily burden, IPFAM without coping subscale may in fact provide more precise and realistic information as a whole diagnostic tool for the actual impact. The fact that the item-level mean score related to coping is 1.6 whereas the other subscales have means of 2.4 – 2.7 show that families are coping extremely well in spite of the impact they report about their child's disability on aspects of their lives seems incongruous. This result may be due to the social and cultural characteristics of the Turkish population. However, the inconsistency related to coping is in concurrence with other studies [2,8]. Also when the values of table 3 are observed the financial consequences of having a child with a disability seems to have the most impact on family life.

Another objective was to show that the Turkish version was a reliable assessment tool for measuring the impact of having a child with developmental disabilities. Two common forms of reliability are internal consistency and test-retest reliability. Internal consistency analysis which refers to the extent to which the measured variance in the score reflects the true score rather than random error yielded good reliability [18]. The Cronbach's Alpha values seen in Table 5 are consistent with and similar to the internal consistency values for the total PACT sample and the Spanish, German and Italian versions of IPFAM [2,15,19]. Similar to the results of a report on PARS III, the reliability estimates for the total impact scores are consistently higher then those for the subscale scores [20]. The fact that coping had the lowest internal consistency is also in concurrence with other studies [2,8].

Tests-retest reliability measures stability over time, by administering the same test to the same subjects at two points in time. In this investigation a time interval of a week was used. A period of one week interval for test- retest reliability studies of parent interviews have been used in other studies [21,22]. We used intra-class correlation coefficient (ICC) to evaluate test-retest reliability from time one to time two. The results of our study showed excellent to good test and retest reliability [23].

Family financial status, educational status, age of child, number of family members may all have an effect on the family burden from a child's disability and it will be interesting to study these effects in large samples. However in this preliminary version study, we chose to investigate the effect of severity of disability by means of two indicators.

Construct validity of the IPFAM was obtained by correlating it with the physiotherapist's evaluation of the severity of the child's disability, and demonstrated good validity [24]. This method is in accordance to the methods utilized in previous studies [6,25-27].

Construct validity of IPFAM was also obtained by correlating it with WeeFIM total score. There is no other study which uses WeeFIM as a construct validity criterion for IPFAM, consequently, we cannot compare our results, but it demonstrates good validity [24]. This correlation between the total score of WeeFIM and the total impact score of IPFAM shows that functional independence of a child may be a reliable indicator of the burden of which the family is subjected to. In other terms, as the functional independence of a child decreases, the impact on the family seems to increase which justifies our pre-mentioned assumption. When the subscales of WeeFIM are correlated with the subscales of IPFAM the strongest relations were found between self-care, mobility, total WeeFIM and total impact.

Translation into different languages and subsequent validation of questionnaires are of importance for international understanding of the measurement properties of these scales. Such studies enable them to be used in different cultural settings and to be utilized with confidence in cross-cultural comparative research trials [1,5,11,12,28]. The effectiveness of comprehensive pediatric outreach programs for youngsters with chronic physical disorders have been tested for their contribution in psychological and social outcomes and long term mental health benefits [29,30] We hope that, in the future, the Turkish version of IPFAM may also be used to evaluate the effects and benefits of social programs aiming to progress family adaptation to community living.

## Conclusion

The results of this study indicate that the Turkish version of the IPFAM is a reliable and valid instrument designed to assess the impact of pediatric developmental disorders on parents HRQoL and family functioning. The subscale inter correlation matrix, total to subscale correlations, test – retest values and the validity measurements all imply that when the coping subscale is excluded, IPFAM becomes a more concise instrument for measuring family impact. The Turkish version of the IPFAM will be further field tested on families who have children with various chronic health conditions and with larger populations of children.

## Competing interests

The authors declare that they have no competing interests.

## Authors' contributions

NB designed the study, worked in all stages of data collection and analysis. IES made substantial contributions to conception and design, worked in all stages of data collection and analysis, wrote the first draft. SE made substantial contributions to conception and design, worked in all stages of data collection, performed the statistical analysis. YY worked in analysis and interpretation of data, revised the manuscript for content. FU made substantial contributions to conception and design, was involved in drafting and revising the manuscript. All authors read and approved the final manuscript.
